# Structural Characterization and Molecular Model Construction of High-Ash Coal from Northern China

**DOI:** 10.3390/molecules28145593

**Published:** 2023-07-23

**Authors:** Benkang Zhu, Xianshu Dong, Yuping Fan, Xiaomin Ma, Suling Yao, Yuanpeng Fu, Ruxia Chen, Ming Chang

**Affiliations:** 1 Department of Mineral Processing Engineering, Taiyuan University of Technology, Taiyuan 030024, China; zhubenkang@126.com (B.Z.); ma_xiaomin@126.com (X.M.); fuyuanpeng@tyut.edu.cn (Y.F.); ruxiachen0828@163.com (R.C.); 18334705322@163.com (M.C.); 2 State Key Laboratory of Mineral Processing, Beijing 100160, China

**Keywords:** high-ash coal, coal structure, molecular simulation, contact angle

## Abstract

High-ash coal, also known as low-grade coal, has becomes a viable alternative in recent years to high-quality coal because available resources have become increasingly scarce due to extensive mining activity. This work aims to provide a comprehensive understanding of the structural characteristics of high-ash coal and construct a plausible molecular structure to elucidate its chemical reactivity in future applications. Its properties were investigated using Solid-state ^13^C nuclear magnetic resonance (^13^C NMR), X-ray photoelectron spectroscopy analysis (XPS), X-ray diffraction (XRD), and Fourier transform infrared spectroscopy (FT-IR). The molecular structure was constructed and validated using Material Studio, LAMMPS Software Package, and MATLAB program. The characterization results revealed that high-ash coal contains 72.15% aromatic carbon, significantly surpassing the percentage of aliphatic carbon (27.85%). The ratio of bridgehead carbon to peripheral aromatic carbon was calculated as 0.67, indicating that the pentacene is the main carbon skeleton form in the high-ash coal structure. Furthermore, oxygen-containing functional groups presented as C=O/O–C–O, C–O, and COO– within the structure along with pyridine and pyrrolic structures. Consequently, the molecular structure comprises pentacene with aliphatic carbon chains, such as methylene, that connect the benzene rings and form a three-dimensional network. The results of a simulated IR spectrum and contact angle simulation aligned with the experimental results, validating the molecular structure of high-ash coal. The chemical formula for the high-ash coal model was determined as C_203_H_189_N_7_O_61_S with a molecular weight of 3734.79.

## 1. Introduction

Around the world, the demand for coal is increasing but the availability of high-quality coal has become increasingly scarce due to extensive mining activity in recent years. To address this issue, the use of high-ash coal, which is categorized as low-grade coal, has gained significant attention not only because it creates economic value, but it also avoids the environmental pollution caused by the large-scale accumulation of coal waste [1]. Therefore, high-ash coal has earned considerable interest in the fields of flotation and gasification. On one hand, it has been used to recover clean coal by flotation in industrial practice [2,3,4] although the ash content, such as quartz and clay minerals, adversely affected the selectivity of the coal [5,6]. On the other hand, some studies showed that high-ash coal was selected as the raw material to produce syngas using pyrolysis [7,8]. Therefore, studying the characteristics of high-ash coal is of significance for understanding its reactivity and promoting clean coal technology.

Understanding the microstructure of coal is conducive to comprehending its reactivity and to exploring its diversity and discrepancy [9,10]. Famous coal models such as those by Wiser, Given, Shinn, and Wender have been used to exhibit the macromolecular structure of raw coal [11,12,13]. However, coal is a complex mixture of organic macerals and rocks, which means that the structure varies because of components and degrees of coalification, especially in different regions. Fortunately, modern material characterization techniques are developing swiftly, making it possible to determine a more accurate chemical structure of a specific kind of coal from a certain area [14]. Feng et al. constructed a coal model from Ningxia province in China using solid-state ^13^C nuclear magnetic resonance (^13^C NMR), X-ray photoelectron spectroscopy (XPS), and Fourier Transform Infrared Spectroscopy (FT-IR). Zhang et al. used a similar method to establish a local lignite model and validated the coal model by comparing the simulated infrared spectrum with that of the experiment [15]. Roberts et al. added X-ray diffraction technology (XRD) and high-resolution transmission electron microscope (HRTEM) analysis to investigate the differences between the inertinite-rich and vitrinite-rich coal in South Africa [16]. The coal samples used in the above research had peculiar properties in either their inner components or coalification degree.

Thanks to the advancements in molecular simulation techniques, it has become possible to establish the spatial configuration of coal structures and explain the reactivity of coal in practical applications. For instance, Guo et al. built a Datong coal structure by combining experimental and simulation methods and performed quantum chemical calculations to optimize the geometric structure of the coal model. The simulated ^13^C-NMR and FT-IR spectra of the established coal model were in good agreement with the experimental results, verifying the accuracy of the coal model [17]. Liu et al. developed a three-dimensional molecular representation of anthracite coal by performing an NMR simulation of the coal structure and comparing the simulated and experimental results to validate the model [18]. Lin et al. investigated the chemical structure of high inertinite coal through quantum chemical simulations. Lin’s group determined the bond length and bond order of the optimum stable geometric configuration of the coal model, which contributed to determining coal reactivity [19]. The construction of coal structures is the initial step for further investigations. Moreover, the enrichment of coal structure knowledge will greatly aid in unraveling the intricate mechanisms behind practical processes such as coal gasification, liquefaction, combustion, and pyrolysis [20,21,22,23]. Zheng et al. used a reasonable coal model to investigate product distributions in brown-coal pyrolysis by reactive molecular dynamic simulations. The results of the study also indicated that the molecular simulation techniques can provide rich clues about coal reactions [24]. Luo et al. selected a coal model to simulate coal char gasification under different atmospheres and provided insight into the reaction pathways of oxygen-functional groups during gasification [25]. Hong et al. investigated the interactions of coal and NH_3_ during co-pyrolysis using reactive molecular dynamic simulations [26]. They found that coal promotes NH_3_ decomposition, while NH_3_ inhibits primary coal pyrolysis. High-ash coal also needs an accurate coal model to enhance in-depth understanding of its chemical reactivity. However, a structure has not been explored comprehensively.

This work aims to provide a comprehensive understanding of the structural characteristics of high-ash coal to construct a plausible molecular structure to elucidate its chemical reactivity in future applications. The structural parameters of high-ash coal were determined using a variety of material characterization techniques: ^13^C NMR, XPS, XRD, and FT-IR. Additionally, the visualization and optimization of the high-ash coal structure were performed using Chemsketch and Material Studio software. To validate the high-ash coal structure, IR spectrum and contact angle simulations were conducted using Material Studio software, LAMMPS Software Package, and MATLAB. This work laid a foundation for future studies and provided new insight into high-ash coal processing, thereby contributing to the advancement of clean coal technology.

## 2. Results & Discussion

### 2.1. Proximate and Ultimate Analysis

The proximate result of the high-ash coal analysis is shown in Table 1. The ash content is 59.67%, indicating it contains many minerals. After the de-ash process, it was reduced to 0.67%, indicating that the acid treatment eliminated most of the minerals. In addition, the moisture of the de-ash sample was 2.87%, and the volatile- and fixed-carbon contents were 38.44 and 58.02%, respectively. The ultimate analysis for the high-ash coal is displayed in Table 2. The sample’s elemental contents of C, H, O, N, and S were 60.26, 2.46, 33.32, 3.38, and 0.58%, respectively. The atomic ratios for H/C, O/C, N/C, and S/C were calculated as 0.49, 0.415, 0.048, and 0.004, respectively, based on proximate analysis. These data will be used to construct the high-ash coal model [27,28].

### 2.2. Solid-State ^13^C Nuclear Magnetic Resonance (^13^C NMR) Analysis

**Figure 1:**
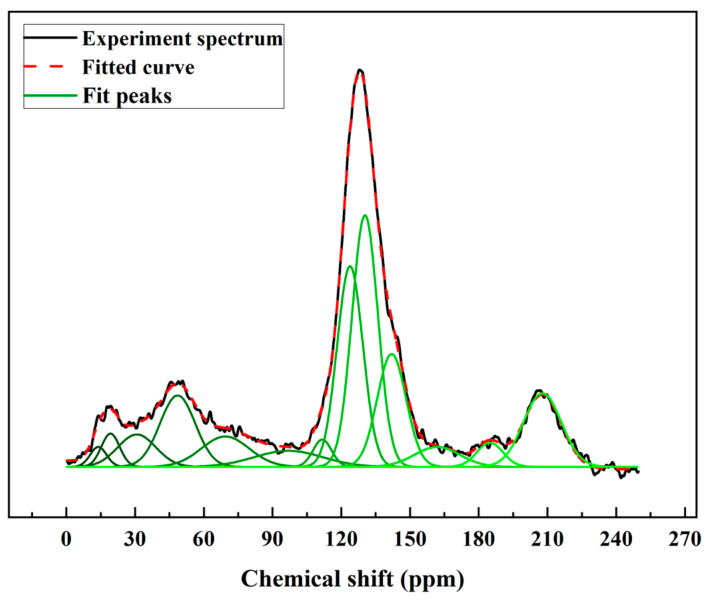
^13^C NMR spectrum and fitting results for the high-ash coal.

The ^13^C NMR spectrum was peak fitted using the Peakfit v4.12 software. The chemical shifts for carbon elements determined the structure attributions. The important parameters of the coal structure were extracted by calculating the peak areas. The ^13^C NMR results are illustrated in Figure 1 and Table 3. Solum et al. proposed 12 important parameters for the coal model based on the ^13^C NMR spectrum [29]. This method was applied to predict the chemical forms of the carbon structure in coal. Therefore, the 12 parameters for high-ash coal were calculated from the ^13^C NMR spectrum and fitting results (Table 4) [24,30].

The results showed the carbon structures of the high-ash coal. In the aromatic part, the content for the bridgehead carbon structures ($f_{a}^{B}$) was 23.93%. The alkylated carbon ($f_{a}^{s}$) and aromatic C bonded to hydroxyl oxygen or ether oxygen parameter ($f_{a}^{P}$) were 12.23 and 3.19%, respectively, indicating the high-ash coal model had alkylated chains distributed in aromatic structures. The protonated and aromatic carbon ($f_{a}^{H}$) indicated the protonated degree of aromatic rings. This value of the high-ash coal was 20.13%. For other aromatic parameters, the non-protonated and aromatic carbon ($f_{a}^{N}$) and aromatic carbon ($f_{a}^{\prime }$) parameters were 39.35 and 59.48%, respectively. These data suggest that the main structure of the high-ash coal was in an aromatic form. For the aliphatic carbon parameters, the methylene C or methine C value ($f_{al}^{H}$) was 14.22%. In addition, the methyl C or quaternary C value ($f_{al}^{*}$) was 3.84%. The parameters for aliphatic carbon bonded to oxygen ($f_{al}^{O}$) value was 9.79%. Furthermore, the total aliphatic carbon values ($f_{al}$) was 27.85%. As for the carbon-contained functional group, the carbonyl group and carboxyl group parameter ($f_{a}^{c}$) was 12.67%, suggesting that there were oxygen-containing functions in the high-ash coal structures. Finally, the high-ash coal’s aromatic carbon value ($f_{a}$) was 72.15%. In light of all the above, the carbon structures of the high-ash coal were revealed, which were subsequently used to build the carbon structures.

Construction work was performed using the 12 parameters of carbon structures above. The structure parameters indicated that the percentage of aromatic carbon was 72.15%, far more than the percentage of aliphatic carbon (27.85%). It is suggested that aromatic carbon was in the majority in the model and that the bridgehead C content was the highest (23.93%), whereas methylene C or methine C (14.22%) was the most common form of aliphatic carbon. Another important parameter $X_{BP},$ representing the ratio of bridgehead to peripheral aromatic carbon, can be calculated by Equation (1) [18,31]:

(1) $$X_{BP}=\frac{f_{a}^{B}}{f_{a}^{H}+f_{a}^{P}+f_{a}^{s}}$$

The $X_{BP}$ value of the sample was 0.67. According to the literature, the $X_{BP}$ of naphthacene is about 0.5, and values for pentacene ranged from 0.57 to 0.67, which was determined by the positions of the benzene rings. It was inferred that the pentacene consisted primarily of the carbon structure.

### 2.3. X-ray Photoelectron Spectroscopy (XPS) Analysis

**Figure 2:**
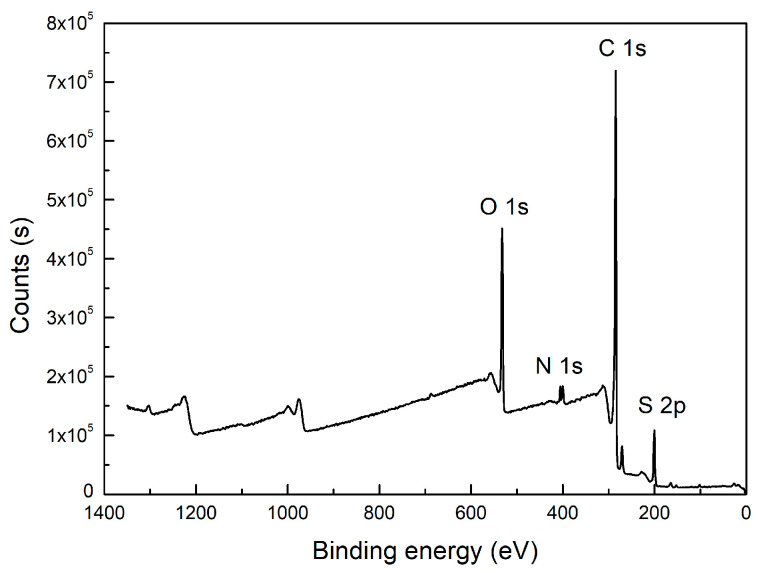
XPS wide scan of the high-ash coal.

**Figure 3:**
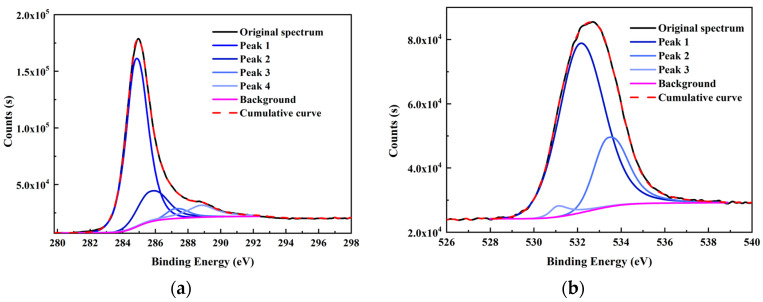

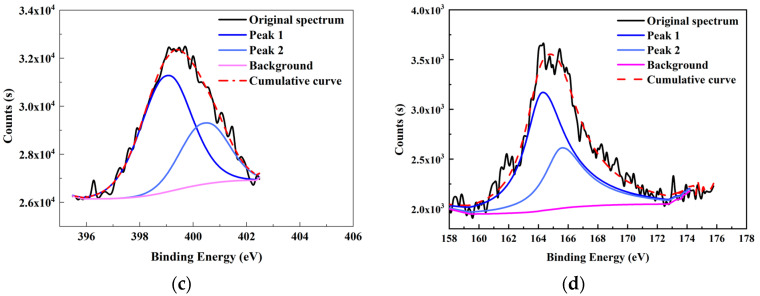
XPS fitting curve of (**a**) C 1s, (**b**) O 1s, (**c**) N 1s, and (**d**) S 2p elements of the high-ash coal.

Besides carbon, heteroatoms such as O, N, and S were also included in the carbon structure. An XPS wide-scan spectrum is shown in Figure 2. It found that the carbon and oxygen peaks were distinct, indicating they were the major elements in the sample. Two weak peaks for nitrogen and sulfur peaks were also observed. The peak-fitting analyses for the four elements were performed using Avantage software. The results are shown in Figure 3. The forms and relative contents of these heteroatoms were analyzed according to the fitting results [10,19].

From the XPS fitting results (Table 5), the C 1s spectrum is attributed to C–C/C–H (284.80 eV), C–O (285.7 eV), C=O (287.23 eV), and COO– (288.82 eV). The relative ratios for the four peaks are 70.08, 16.99, 4.27, and 8.66%, respectively. It was found that the C–C/C–H carbon form was the majority in the spectrum. In addition, the O 1s spectrum contained three peaks—C=O/O–C–O (531.08 eV), C–O (532.13 eV), and COO– (533.41 eV)—with relative percentages of 2.43, 74.09, and 3.48%, respectively. N 1s spectrum contained two prominent peaks: pyridine (398.99 eV) and pyrrolic (400.38 eV) [32]. The calculated pyridine and pyrrolic contents were 67.40 and 32.60%, respectively. Lastly, the S 2p spectrum can be divided into two peaks of mercaptan thiophenol (164.27 eV) and thiophene (165.57 eV), and the relative contents were 66.16 and 33.84%, respectively. However, the S element content was shallow from the ultimate analysis (0.58%); hence, the corresponding number of S structures was also limited. All the data above will be used later to design the high-ash coal.

### 2.4. X-ray Diffraction (XRD) Analysis

**Figure 4:**
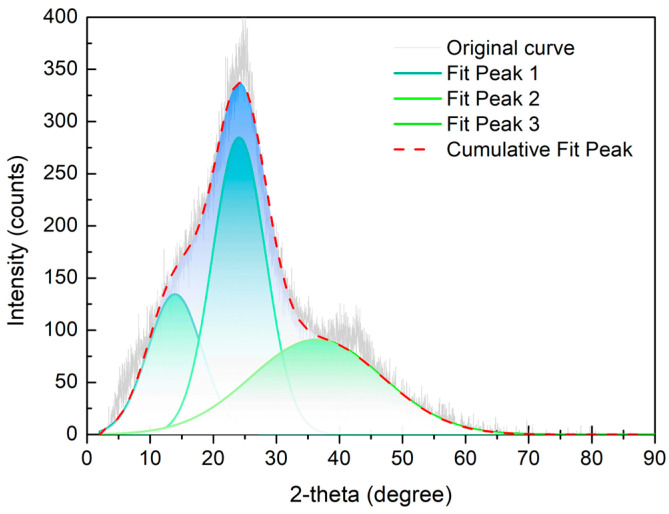
XRD results and fitted curve of the high-ash coal.

The XRD pattern of the high-ash coal is shown in Figure 4. The original pattern contained two peaks: a (002) peak with a 24° diffraction angle, and a (100) peak between 35 and 50° [33]. The (002) peak was composed of the (002) peak and γ peak bands. Previous literature had said that the (002) peak band represented the diffraction signal for the aromatic layer in coal, and the γ peak band reflected the signal for the aliphatic carbon structure [16,30]. Moreover, the (100) peak revealed the degree of stacking in the aromatic lamellae in the coal sample. The fitting work for the XRD curve was completed by Origin 8.5 Pro software. According to the fitting data, aromatic layer distance ($d_{002}$), stacking height of aromatic layer ($L_{c}$), diameter of aromatic layer ($L_{a}$), average number of the aromatic layer ($N_{ave}$), and aromaticity were calculated using Equations (2–6) [34,35].

(2) $$d_{002}=\frac{\lambda }{2sin\theta_{002}}$$

(3) $$L_{c}=\frac{0.94\lambda }{\beta_{002}cos\theta_{002}}$$

(4) $$L_{a}=\frac{1.84\lambda }{\beta_{100}cos\theta_{100}}$$

(5) $$N_{ave}=\frac{L_{c}}{d_{002}}$$

(6) $$f_{a}=\frac{A_{002}}{A_{002}+A_{\lambda }}$$

where λ is the X-ray wavelength (1.54 Å in this case); $\theta_{002}$ and $\theta_{100}$ are the diffraction angles for the (002) and (100) peak bands, respectively; $\beta_{002}$ and $\beta_{100}$ are the half-maximum intensities for the (002) and (100) peak bands, respectively; and $A_{002}$ and $A_{\lambda }$ are the fitted areas for the (002) and λ peak bands, respectively.

Parameters extracted from the XRD pattern are displayed in Table 6. From the fitting results of the (002) peak, it was suggested that the diffraction angles for the (002) and λ peak bands were 13.93° and 24.09°, respectively. The layer distance for the aromatic layer was 3.69 Å; the stacking height of the aromatic layer was 8.54 Å; and the average number of the aromatic layer was 2.31. The parameters for the (100) peak indicated that the diameter of the aromatic layer was 6.97 Å. More importantly, the aromaticity value calculated from the XRD analysis was 0.68, which was very close to the value from ^13^C NMR. Therefore, the ratio of aromatic carbon appeared to be much higher than that of aliphatic carbon in the high-ash coal.

### 2.5. High-Ash Coal Model Construction

The chemical structure for the high-ash coal was sketched based on the characterization analysis. The main carbon structures were determined according to the ^13^C NMR analysis results. The types and numbers of aromatic carbon structures are illustrated in Table 7. It was determined that the aromatic carbon structures included five pentacene, one naphthacene, four pyridine, and three pyrrolic structures. The $X_{BP}$ in this scenario was 0.657, which was close to the ^13^C NMR experiment result (0.67). Then the total carbon numbers were determined with the element analysis and ^13^C NMR results. After that, the XPS analysis helped determine the types and numbers of aliphatic carbon and other functional groups. Thus, for aliphatic carbon, there were nine methine or quaternary carbon structures, 35 methene, and 31 carboxyl and carbonyl groups. The S atom content was very low, so only one mercaptan thiophenol was added into the model. Therefore, the chemical formula for the high-ash coal model was determined to be C_203_H_189_N_7_O_61_S with a molecular weight of 3734.79.

### 2.6. Model Configuration Optimization

**Figure 5:**
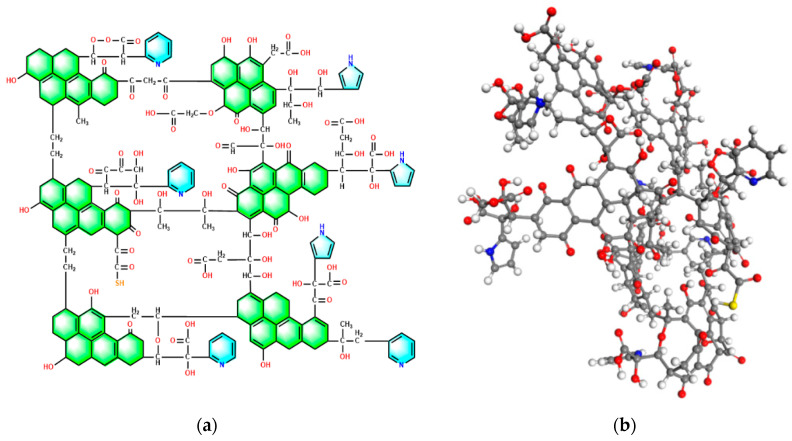
High-ash coal model plane structure (**a**) and configuration after optimization (**b**). (Grey ball: C atom, red ball: O atom, white ball: H atom, blue ball: N atom, yellow ball: S atom).

The plane carbon model was converted to a 3D form in Material Studio 2019 software, and the following step, configuration after optimization [36,37], is shown in Figure 5. Geometry optimization and molecular dynamic simulation were performed to derive the energy minimum configuration. Some parts were seen to suffer from many changes: some bonds were bent or twisted after simulation, leading to changes in bond length and angle. The model was displayed in a 3D network structure. The stacking structure of the aromatic layer was apparent, which conformed to the XRD results. Moreover, many oxygen-containing structures were distributed in the fringe of the aromatic layer, most of which were directly connected with carbon atoms. The pyridine and pyrrolic structures were also allocated in the structure, aligning with XPS analysis. Therefore, the built high-ash coal structure is consistent with the characterization results.

### 2.7. Model Validation

#### 2.7.1. FT-IR Experiment and Simulation Results

**Figure 6:**
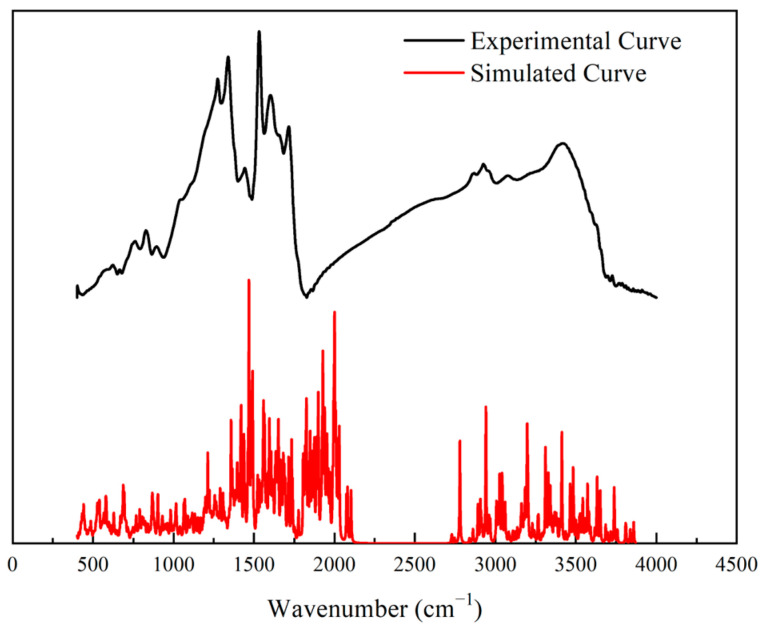
Experimental and Simulated IR spectrum.

The FT-IR spectrum was used to validate the constructed model as shown in Figure 6. The spectrum had four main regions—700–900, 1000–1800, 2700–3000, and 3000–3500 cm^−1^—with the corresponding aromatic structures, oxygen-containing groups, aliphatic carbon structures, and hydroxide radical groups, respectively. Specifically, the peak of 828.92 cm^−1^ represented benzene’s tri- and tetra- substitution. Many peaks were found in the range of 1000–1800 cm^−1^, indicating abundant oxygen-containing groups in the structure. Among these peaks, the 1274 cm^−1^ peak denoted a C–O structure in a phenoxy structure, the 1339 and 1442 cm^−1^ peaks signified the CH_2_/CH_3_ vibration; the 1602 and 1532 cm^−1^ peaks were the signals for the C=C structure in the aromatic nucleus; and the 1716 cm^−1^ peak represented the vibration of the C=O structure. Moreover, the peak at 2925 cm^−1^ implied –CH_3_ structures, and the peak at 3419.68 cm^−1^ was attributed to –OH structures [38,39].

The simulated IR spectrum was introduced to validate the high-ash coal model. Its frequency calculation was performed using the VAMP module in Materials Studio 2019 software. By comparison, the experimental and simulated IR spectra were consistent, apart from the discrepancies caused by the different resolutions of the experiment and the calculation. Specifically, the two spectra were in good agreement in the range of 1000–1800 and 3000–3500 cm^−1^, indicating the distributions of oxygen-containing groups and hydroxy groups in the chemical structure were reasonable. Therefore, the accuracy of the model was verified by the IR spectra.

#### 2.7.2. Contact Angle Measurement and Simulation Results

**Figure 7:**
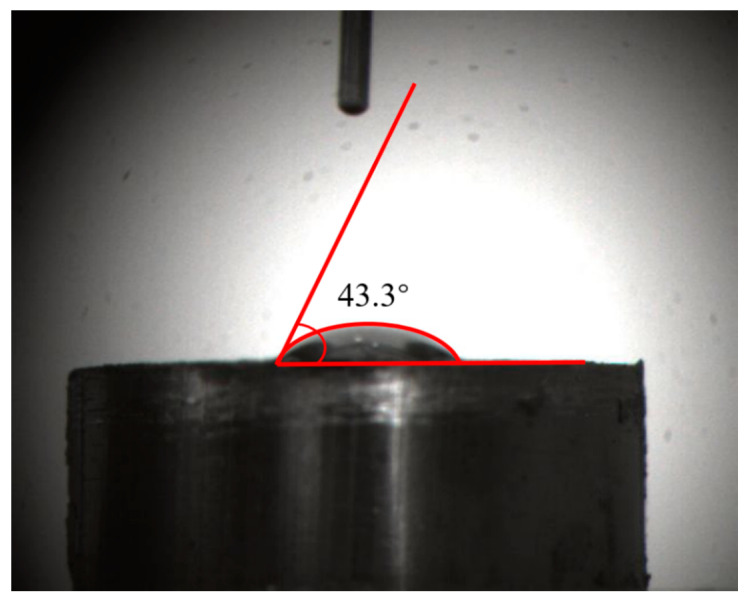
Contact angle measurement of the high-ash coal.

To evaluate the hydrophobicity of the surface of the high-ash coal model further, contact angle measurement and simulation work were performed. The contact angle measurement was implemented using the Contact Angle Measurement Instrument produced by Beijing Hake Apparatus Corporation, China. In the contact angle measurement, 1 g coal powder was loaded into a tablet pressing mold. The powder was compacted and shaped into a circular slice under 15 MPa for 5 min. After that, the circular slice was obtained. Then a droplet of water onto the coal surface formed a stable shape. The contact angle value was measured by analyzing software developed by Beijing Hake Apparatus Corporation, China. The measurement result was 43.3° using the ancillary image process software of the measurement instrument (see Figure 7). It was suggested that the surface of the high-ash coal model exhibited relatively strong hydrophilicity, indicating that there should be hydrophilic groups on the surface. From the carbon model configuration depicted in Figure 5, hydroxyls, carbonyls, and carboxyl were distributed on the surface of the coal model, which aligned with the measurement results.

**Figure 8:**
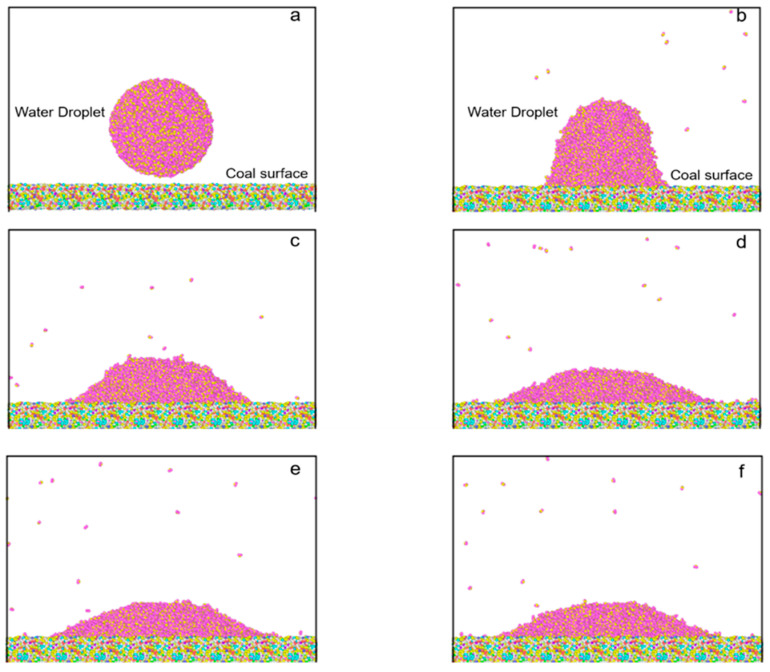
Configuration of water droplet on model surface at different times: (**a**) 0 ps, (**b**) 50 ps, (**c**) 100 ps, (**d**) 500 ps (**e**) 1000 ps, (**f**) 1500 ps.

In the simulation part, Figure 8 selects 6 plots at different times in the process to compare the variation of the droplet configurations. The figures show that the original water droplet tended to spread out on the surface with time changes. At 50 ps, many water molecules fell onto the coal surface. When the time came to 100 ps, almost all water molecules moved to the surface, forming an ambiguous water–coal interface. At 500 ps, the motion of water molecules became gradually slower and nearly stabilized. Finally, an obvious interface of the water droplet and the coal surface was produced and used for contact angle calculation.

**Figure 9:**
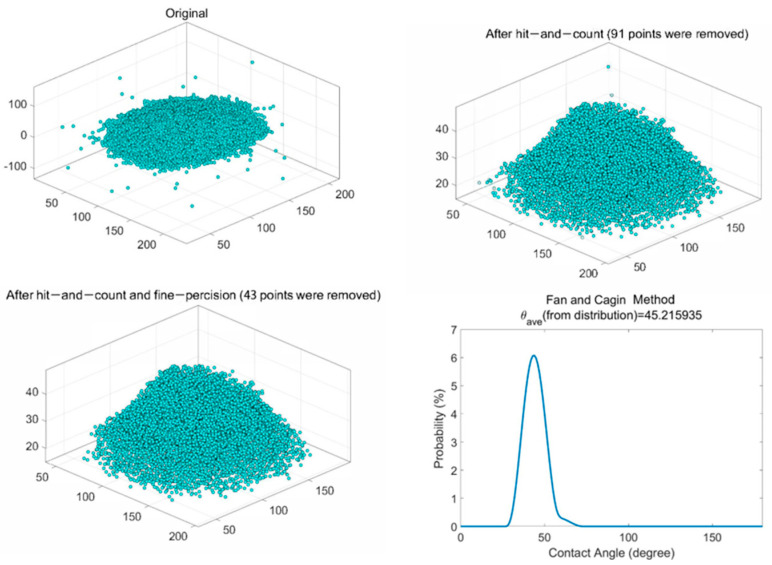
Contact angle calculation process: (**a**) original droplet configuration, (**b**) configuration after hit−and−count step, (**c**) configuration after hit−and−count and fine−precision, (**d**) contact angle calculation by the Fan and Caign method.

In the data processing part, the stabilized configuration of the water droplet was loaded into MATLAB to calculate the contact angle. Figure 9a shows the original water droplet model. Khalkhali’s method identified the water droplet first and eliminated the outliers (water molecules escape from the droplet) to create a convex hull [40]. Figure 9b,c displays the water droplet dataset after removing the outliers. Finally, the contact angle was calculated by Fan and Caign’s method (Figure 9d) [41]. The average contact angle was 45.2°, close to the measurement result (43.3°). Therefore, the consistent result indicated that the high-ash coal model had reasonable functional groups on the surface, again validating the constructed model.

## 3. Method

The high-ash coal sample used in this study underwent an initial preprocessing step involving an HCl–HF–HCl acid treatment. Following this, various analytical techniques were employed to gather structural information about the sample: Solid-state ^13^C nuclear magnetic resonance (^13^C NMR), X-ray photoelectron spectroscopy analysis (XPS), X-ray diffraction (XRD), and Fourier transform infrared spectroscopy (FT-IR). The ^13^C NMR test provided insight into the presence of aromatic and aliphatic carbon forms within the high-ash coal structure. XPS analysis facilitated the determination of the distribution of functional groups, such as carboxyl and pyridine groups. The XRD pattern contained mainly the information of the stacking degree in the aromatic lamellae in the high-ash coal. The FT-IR spectrum was scanned to identify the functional structures in the high-ash coal. Moreover, the experimental FT-IR spectrum was compared with simulation results to validate the high-ash coal model. To analyze the chemical structure of the high-ash coal further, ChemSketch software and Material Studio 2019 software were used for plotting and visualizing purposes. To validate the high-ash coal model, FT-IR and contact angle simulation were carried out using Material Studio 2019 software, LAMMPS Software Package, and the MATLAB program.

### 3.1. Experiment and Methodology

#### 3.1.1. Sample Collection and Acid Treatment

The high-ash coal sample was collected from a coking coal preparation plant in Shanxi Province in northern China. The sample was dried, ground, and put through a 0.074 mm sieve. Then a proximate analysis was performed according to the Chinese national standard (GB/T 212-2008). The results (Table 1) showed a high-ash content of 59.67%. Hence, a HCl–HF–HCl acid treatment was adopted to reduce the mineral content.

The high-ash coal sample was stirred in a 5 mol/L aqueous HCl solution with a mass ratio of 1:5 for 5 h at 60 °C, after which ultrapure water was used to rinse the sample until the pH reached 7. Then the sample was dried in an oven at a temperature below 60 °C. Next, the dried sample was leached again using 40% HF and 5 mol/L HCl solution with the same test condition and treatment process. After rinsing and drying, the minerals in the high-ash coal were eliminated.

#### 3.1.2. Characterization Analysis

The ultimate analysis was performed using a Vario EL Cube Elementar elemental analyzer. The ^13^C NMR analysis was performed using Bruker Avance II 400M solid-state spectrometer equipped with a 4 mm MAS probe. The cross-polarization (CP) magic angle spinning (MAS) experiment was applied. The standard CP MAS was used to suppress the sideband with a contact time of 0.05 s, the magic angle rotation speed was 10 kHz, and the recycle delay time was 4 s [27]. The XPS analysis was examined by a Thermo ESCALAB 250XI spectrometer. The parameters were Al Kα radiation, working voltage 12 kV, electric currency current 6 mA, pass energy 150 eV with a step size of 1 eV. The binding energy was corrected by the C 1 s hydrocarbon peak at 284.80 eV [42]. The XRD spectrum was measured using a MiniFlex600 crystalline diffractometer from RIGAKU company. The test condition was Cu–Kα radiation; tube pressure was 40 kV; electric current was 15 mA, and scanning range was 5–100° with a speed of 2°/min [43].

### 3.2. Model Construction Methods

The plane structure of the high-ash coal model was plotted by ChemSketch software. Then the 3D structure was visualized in Material Studio 2019 software and optimized by molecular mechanics and dynamics to derive the minimum energy configuration [14,20]. The molecular mechanics’ optimization task was performed in the Forcite module in the software. The Dreiding force field was chosen, and the calculation accuracy was ultra-fine. The algorithm was smart minimization, and the maximum iterations were 10,000 steps. After the molecular mechanic optimization, annealing molecular dynamics was also performed in the Forcite module. The calculation accuracy was set as fine, and the annealing cycle was 10. The temperature was controlled by a Nose program ranging from 300 to 900 K. The NVT ensemble was applied, and the total number of the steps was 10,000. The total time step was set as 1 fs [16].

### 3.3. Model Validation

#### 3.3.1. FT-IR Simulation

The FT-IR analysis was performed to study the surficial group of samples. According to peak position and height, the types and numbers of the chemical structure were summarized. The frequency calculation was performed using the VAMP module in MS 2019 software. The energy task was selected, the Hamiltonian was NDDO and AM1; multiplicity was Auto; and spin was RHF. The convergence scheme was determined as standard, and the precision was fine. After the calculation was finished, the simulated IR spectrum was acquired using the vibration analysis in Materials Studio 2019. Finally, a comparison of the results between the experimental and simulated IR spectra contributed to the model validation.

#### 3.3.2. Contact Angle Simulation

Contact angle simulation work had three steps: model construction, simulation running, and data processing. The first step was to build a model showing a water droplet on the coal surface. First, a water droplet containing 8985 water molecules was constructed using the Forcite module in Material Studio software. Then the droplet was optimized using PCFF_INTERFACE forcefield [44], and a water sphere was established. After that, eight coal molecules were pressed by two graphite layers to produce a flat coal surface [45,46]. The thickness of the compressed coal model was 18 Å, and the surface sizes were 30 × 28 Å. Subsequently, a supercell of coal surface with 240 × 224 Å was constructed. Finally, the water droplet was placed on the 5 Å top of the coal surface, forming the original configuration for the contact angle simulation.

The second step was to run the program by the LAMMPS Software Package [47]. The program was set as 1500 ps with the condition at 298 K and 1 atom. The PCFF_INTERFACE forcefield was also used in this part. One trajectory file was exported every 1000 steps to be read by Ovito software to manifest the simulation.

The third step of contact simulation, data processing, was the most crucial. The stabilized configuration of the water droplet was loaded into MATLAB to calculate the contact angle. The data process mode referred to Khalkhali’s method [40], which had two procedures: identification of the liquid droplet and convex hull creation. The hit-and-count method was applied to eliminate the outliers (water molecules escaped from the droplet). Finally, the average contact angle was calculated by Fan and Caign’s equations [41].

## 4. Conclusions

A comprehensive characterization of high-ash coal was conducted to construct its chemical structure. Its properties were investigated using various material characterization techniques: ^13^C NMR, XPS, XRD, and FT-IR. It was found that the high-ash coal consisted of 72.15% aromatic carbon, which significantly exceeded the percentage of aliphatic carbon (27.85%). The ratio of the bridgehead carbon to peripheral aromatic carbon was calculated as 0.67, indicating that the pentacene was the main carbon skeleton form within the high-ash coal structure. Moreover, oxygen-containing functional groups presented as C=O/O–C–O, C–O, and COO–, accounting for 2.43, 74.09, and 3.48%, respectively. Pyridine and pyrrolic structures existed in relative concentrations of 67.40 and 32.60%, respectively. Furthermore, the layer distance for the aromatic layer was determined to be 3.69 Å; the stacking height of the aromatic layer was 8.54 Å; and the average number of the aromatic layer was 2.31. The established formula of the high-ash coal model was C_203_H_189_N_7_O_61_S having a molecular weight of 3734.79. The optimized configuration of the model presented a 3D network in which pentacene constituted the carbon skeleton, and aliphatic carbon chains connected the scattered carbon skeletons. The simulated IR spectrum was in good agreement with the experimental one, validating the accuracy of the built model. Contact angle simulation and calculation were performed by LAMMPS Software Package and MATLAB, and the calculated result was 45.3°, which closely matched the measured result of 43.3°, further validating the high-ash coal structure. This work contributes to comprehending the microstructure and reactivity of high-ash coal from Shanxi Province in northern China and lays a foundation for reactive high-ash coal mechanism studies at an atomic level.

## Figures and Tables

**Table 1:** Proximate analysis results of high-ash coal.

Sample	Proximate Analysis (wt %)			
	M_ad_	A_ad_	V_ad_	FC_ad_
High-ash coal	2.69	59.67	13.31	24.33
High-ash coal after acid treatment	2.87	0.67	38.44	58.02

ad, air-dried.

**Table 2:** Ultimate analysis results of the high-ash coal.

Sample	Ultimate Analysis (daf, wt %)				
	C	H	O *	N	S
High-ash coal	60.26	2.46	33.32	3.38	0.58

daf, dry and ash-free; *, by difference.

**Table 3:** Fitting parameters and structure attribution from ^13^C NMR for high-ash coal.

Chemical Shifts (ppm)	Structure Attribution	Relative Area (%)
0–15	Aliphatic methyl (R–CH_3_)	1.24
15–26	Aromatic methyl (Ar–CH_3_)	2.60
26–37	Methene (CH_2_)	4.32
37–50	Methine or quaternary carbon (C, CH)	9.90
50–56	Oxy-methyl/methene (O–CH_3_, O–CH_2_)	4.61
60–70	Oxy-methine	3.51
75–90	Oxy-quaternary carbon	1.67
95–124	Protonated and aromatic carbon (Ar–H)	20.13
124–137	Aromatic bridgehead carbon (C–C)	23.93
137–149	Aliphatic substituted aromatic carbon (Ar–C)	12.23
149–164	Oxy-aromatic carbon (Ar–O)	3.19
165–195	Carboxyl group (COOH)	2.48
195–220	Carbonyl group (C=O)	10.19

**Table 4:** Structural parameters of high-ash coal.

Index	$f_{a}$	$f_{a}^{c}$	$f_{a}^{\prime }$	$f_{a}^{N}$	$f_{a}^{H}$	$f_{a}^{P}$	$f_{a}^{s}$	$f_{a}^{B}$	$f_{al}$	$f_{al}^{*}$	$f_{al}^{H}$	$f_{al}^{O}$
High-ash coal	72.15	12.67	59.48	39.35	20.13	3.19	12.23	23.93	27.85	3.84	14.22	9.79

*f_a_*, total aromaticity, $f_{a}^{c}$, carbonyl group and carboxyl group C; $f_{a}^{\prime }$, aromatic C; $f_{a}^{N}$, non-protonated and aromatic C; $f_{a}^{H}$, protonated and aromatic C; $f_{a}^{P}$, aromatic C bonded to hydroxyl oxygen or ether oxygen; $f_{a}^{s}$, alkylated aromatic C; $f_{a}^{B}$, aromatic bridgehead C; $f_{al}$, total aliphatic C; $f_{al}^{*}$, methyl C or quaternary C; $f_{al}^{H}$, methylene C or methine C; $f_{al}^{O}$, aliphatic C bonded to oxygen.

**Table 5:** Curve fitting results and percentage calculation from XPS.

Element Peak	Binding Energy (eV)	Group Attribution	Percentage (%)
C 1s	284.80	C–C/C–H	70.08
	285.70	C-O	16.99
	287.23	C=O/O–C–O	4.27
	288.82	COO-	8.66
O 1s	531.08	C=O/O-C–O	2.43
	532.13	C-O	74.09
	533.41	COO–	23.48
N 1s	398.99	Pyridine nitrogen	67.40
	400.38	Pyrrolic nitrogen	32.60
S 2p	164.27	Mercaptan thiophenol	66.16
	165.57	Thiophene	33.84

**Table 6:** Calculated indices from XRD results.

Index	$2\theta_{\lambda }({}^{\circ})$	$2\theta_{002}({}^{\circ})$	$2\theta_{100}({}^{\circ})$	$d_{002}(Å)$	$L_{c}$ (Å)	$L_{a}$ (Å)	$N_{ave}$	$f_{a}$
Sample	13.93	24.09	36.39	3.69	8.54	6.97	2.31	0.68

**Table 7:** Types and numbers of aromatic structures in the high-ash coal model.

Types	Number
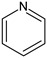	4
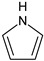	3
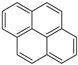	1
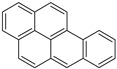	5

## Data Availability

Not applicable.
